# Analysis of prognostic factors in localized high-risk prostate cancer patients treated with HDR brachytherapy, hypofractionated 3D-CRT and neoadjuvant/adjuvant androgen deprivation therapy (trimodality therapy)

**DOI:** 10.1093/jrr/rrt134

**Published:** 2013-12-17

**Authors:** Manabu Aoki, Kenta Miki, Masahito Kido, Hiroshi Sasaki, Wataru Nakamura, Yoshikazu Kijima, Masao Kobayashi, Shin Egawa, Chihiro Kanehira

**Affiliations:** 1 Department of Radiology, The Jikei University School of Medicine, 3-25-8 Nishi-Shimbashi, Minato-Ku, Tokyo 105-8461, Japan; 2 Department of Urology, The Jikei University School of Medicine, 3-25-8 Nishi-Shimbashi, Minato-Ku, Tokyo 105-8461, Japan

**Keywords:** high-risk prostate cancer, HDR, trimodality therapy, PSA response

## Abstract

Trimodality therapy consisting of high dose rate (HDR) brachytherapy combined with external beam radiation therapy (EBRT), neoadjuvant hormonal therapy (NHT) and adjuvant hormonal therapy (AHT) has been used to treat localized high-risk prostate cancer. In this study, an analysis of patients receiving the trimodality therapy was performed to identify prognostic factors of biochemical relapse-free survival (bRFS). Between May 2005 and November 2008, 123 high-risk prostate cancer patients (D'Amico classification) were treated with NHT prior to HDR brachytherapy combined with hypofractionated EBRT. Among these patients, 121 had completed AHT. The patients were assigned by time to be treated with a low-dose or high-dose arm of HDR brachytherapy with subsequent hypofractionated 3D conformal radiation therapy (3D-CRT). Multivariate analysis was used to determine prognostic factors for bRFS. With a median follow-up of 60 months, the 5-year bRFS for all patients was 84.3% (high-dose arm, 92.9%; low-dose arm, 72.4%, *P* = 0.047). bRFS in the pre-HDR PSA ≤ 0.1 ng/ml subgroup was significantly improved compared with that in the pre-HDR PSA > 0.1 ng/ml subgroup (88.3% vs 68.2%, *P* = 0.034). On multivariate analysis, dose of HDR (*P* = 0.045, HR = 0.25, 95% CI = 0.038–0.97) and pre-HDR PSA level (*P* = 0.02 HR = 3.2, 95% CI = 1.18–10.16) were significant prognostic factors predicting bRFS. In high-risk prostate cancer patients treated with the trimodality therapy, the dose of HDR and pre-HDR PSA were significant prognostic factors. The pre-HDR PSA ≤ 0.1 subgroup had significantly improved bRFS. Further studies are needed to confirm the relevance of pre-HDR PSA in trimodality therapy.

## INTRODUCTION

There are many options for radical radiotherapy for localized prostate cancer. In addition to conventional external beam radiation therapy (EBRT), such as 3D conformal radiotherapy (3D-CRT) and intensity modulated radiotherapy (IMRT), brachytherapy, such as low-dose rate (LDR) or high-dose rate (HDR) brachytherapy, is performed. While efficacy has been reported for all modalities in low-to intermediate-risk prostate cancer, it has been reported that the efficacy of monotherapy with radiation in high-risk prostate cancer is limited [1].

There have been many reports that dose escalation is correlated with PSA-free survival in prostate cancer, and it is known that the contribution of dose escalation is more marked in higher risk prostate cancer [1]. In current radiotherapy for high-risk prostate cancer by EBRT, IMRT is primarily used to increase the dose level and reduce adverse events. In brachytherapy, biologically more effective HDR brachytherapy is used in prostate cancer, because prostate tumors have a lower α/β ratio than the surrounding normal tissue, and it has been reported worldwide that this modality is very effective [2, 3].

Based on the results of long-term adjuvant hormonal therapy (AHT) as multimodality treatment for high-risk prostate cancer reported by the EORTC [4], long-term (2- to 3-year) hormonal therapy has become standard treatment in radiotherapy for high-risk prostate cancer. However, the dose level as the curative dose for high-risk prostate cancer in the EORTC randomized controlled trial (RCT) was relatively low. In Japan, neoadjuvant hormonal therapy (NHT), radical radiotherapy with dose escalation, and AHT are generally accepted treatment for high-risk localized prostate cancer. For NHT, the efficacy was discussed in two reports from Canadian trials [5, 6], but no consensus has been reached on the optimal duration of treatment. We have taken a trimodal approach to treat high-risk localized prostate cancer, combining NHT, HDR brachytherapy, 3D-CRT and AHT since May 2005. This study has analyzed the results of patients receiving the trimodality therapy from May 2005 to November 2008 to identify prognostic factors for biochemical relapse-free survival (bRFS).

## MATERIALS AND METHODS

A total of 123 high-risk localized prostate cancer patients who received NHT, HDR brachytherapy, 3D-CRT and AHT between May 2005 and November 2008 were included in the study. Follow-up was commenced at the initiation of HDR, and follow-up of the last patient with trimodality therapy started in November 2008. The D'Amico classification system was used to classify the risk. The median age of patients was 69 years (range: 59–82), and the median initial PSA was 23.5 ng/ml (range: 3.9–365). The Gleason score (GS) was 6, 7, 8, 9 and 10 in 10, 32, 42, 32 and 7 patients, respectively, with a median score of 8. The median follow-up was 60 months (range: 26–88), and the median duration of NHT was 8 months (range: 1–29). The median duration of AHT was 24 months (range: 3–24), and 98% of patients received AHT for 24 months (Table 1). NHT and AHT included total androgen blockade (TAB) in 86 patients (4–22 months) and LH-RH alone in 22 patients (6–18 months). Neoadjuvant chemotherapy prior to radiotherapy included DTX in three patients (6 months), TAB + DTX in seven patients (7–16 months), and LH-RH + DTX in five patients (10–12 months). No patient received adjuvant chemotherapy after radiotherapy. Details of endocrine therapy and chemotherapy are shown in Table 2. After neoadjuvant therapy, all patients received HDR Brachytherapy (HDR-BT), followed by hypofractionated 3D-CRT to the prostate and seminal vesicle at a mean interval of 10 days.

**Table 1.** Patient characteristics

	Low-dose arm (*n* = 74)	High-dose arm (*n* = 49)	*P*-value
**Median follow up** (months)	60 (34-88)	60 (26-72)	0.31
**Age (median)** (years)	68 (59–81)	70 (52–82)	0.45
**Initial PSA (median)** (ng/ml)	20.1 (3.9–192)	25 (4.17–365)	0.5
	GS 6: 6 pts	GS 6: 4 pts	
	GS 7: 19 pts	GS 7: 13 pts	
**Gleason score**	GS 8: 25 pts	GS 8: 17 pts	0.67
	GS 9: 21 pts	GS 9: 11 pts	
	GS 10: 3 pts	GS 10: 4 pts	
**Duration of NHT**			
Median duration of NHT	8 (1–22)	10 (6–29)	
NHT ≤ 6 months	23 pts	3 pts	0.082
6 months < NHT ≤ 12 months	35 pts	34 pts	
NHT > 12 months	16 pts	12 pts	
Median duration of AHT (months)	24 (3–24)	24 (all cases 24)	

GS = Gleason score, pts = patients, NHT = Neoadjuvant Hormonal Therapy, AHT = Adjuvant Hormonal Therapy.

**Table 2.** Types of neo-adjuvant hormonal therapy and chemotherapy

	Low dose arm (*n* = 74)	High dose arm (*n* = 49)
TAB	45	41
LH-RH	18	4
TAB + DTX	4	3
LH-RH + DTX	4	1
DTX	3	0

TAB = Total Androgen Blockade, LH-RH = LH-RH agonist, DTX = Dosetaxel.

Prior to treatment, each patient underwent lumbar spinal anesthesia and epidural anesthesia with placement of a Foley catheter. The patient was placed into the lithotomy position. Needle and Au marker placement were then performed with TRUS guidance. The patient underwent a CT scan for planning using the computer. The number of needles amounted to ∼ 15–20. The patient was given HDR-BT twice within a couple of days using MicroSelectron^®^.

In prostate HDR brachytherapy, the planning target volume (PTV), which was the gross target volume (GTV = prostate) with a 4-mm margin around the prostate (except 2-mm margin only on the rectum) was treated at the prescribed dose. The total dose of HDR brachytherapy consisted of two arms: a low-dose arm in the early study period and a high-dose arm in the late study period. In the low-dose arm, the above-mentioned PTV was treated twice in a day with a first dose of 5 Gy and a second dose of 6 Gy (at an interval of 6 h). We administered 5 Gy, 6 Gy to the PTV. The maximum urethral dose was kept to 150% of the prescription dose. In the high-dose arm, a total dose of 18 Gy was administered once daily for 2 d (9 Gy × 2 fx). In a 3D-CRT setting, the PTV—defined as the GTV (prostate and seminal vesicle) with a 5-mm margin—plus an additional 5-mm PTV margin was treated at the prescribed dose. Combined 3D-CRT was administered at total dose of 45 Gy (3 Gy/fx) for the low-dose arm and 40 Gy for the high-dose arm (2.5 Gy/fx). PSA failure following trimodality therapy was defined according to the Phoenix/ASTRO definition. We compared the two arms in terms of population by Mann-Whitney's U tests. bRFS's were compared with the Kaplan–Meyer method and log rank statistics. Multivariate analysis was performed to analyze prognostic factors in this study using SPSS ^®^ and STATFLEX ^®^.

This study was conducted in accordance with the Helsinki Declaration and the intramural ethics committee. Written informed consent was obtained prior to radiotherapy.

## RESULTS

**Fig. 1.**
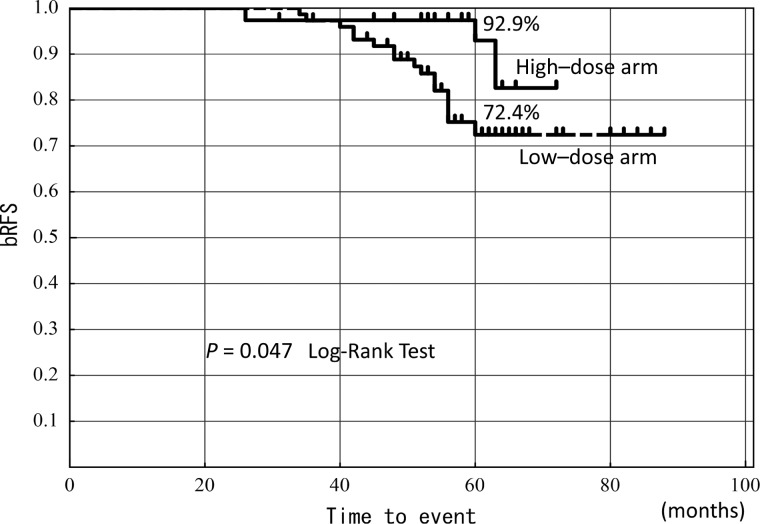
5-year bRFS by HDR dose.

**Fig. 2.**
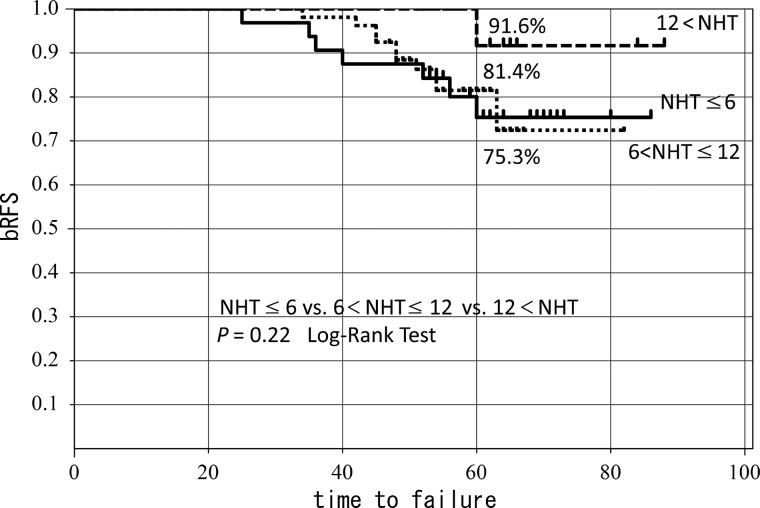
5-year bRFS by duration of NHT.

The median duration of follow-up was 60 months, and the 5-year bRFS was 84.3% in the entire population. The 5-year bRFS was 92.9% in the high-dose arm, and 72.4% in the low-dose arm (*P* = 0.047) (Fig. 1). The 5-year bRFS did not significantly differ in the i-PSA level (<20 ng/ml: 89.9% vs ≥ 20 ng/ml : 79.1%) (P = 0.20). In the GS arms, the 5-year bRFS also did not significantly differ between any of the arms (GS 6–7 : 92% vs GS 8 : 83.6% vs GS 9–10: 65.8%, *P* = 0.09). The treatment outcome in the patients stratified by the duration of NHT into three arms (NHT ≤ 6 months, 6 months < NHT ≤ 12 months, and 12 months < NHT) is shown in Fig. 2. The 5-year bRFS was 75.3%, 81.4% and 91.6%, respectively. A statistically significant difference could not be observed between any of the arms (*P* = 0.2).

**Fig. 3.**
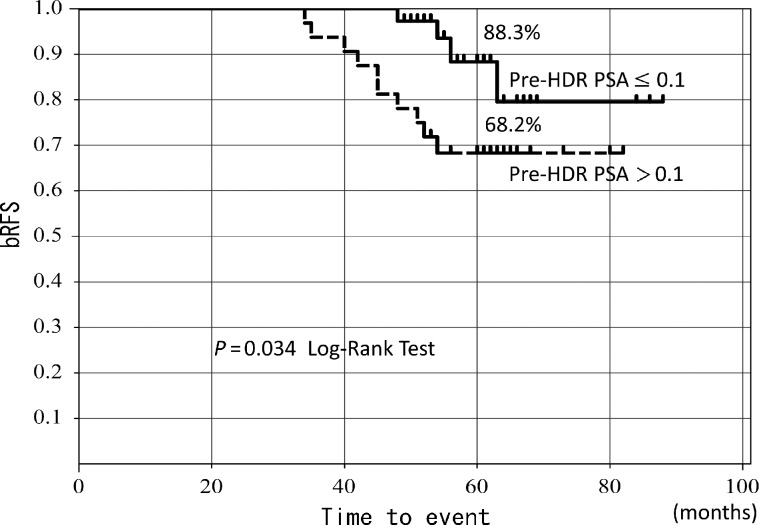
5-year bRFS by pre-HDR PSA level.

The treatment outcome for the patients stratified by the PSA value (pre-HDR PSA) immediately before HDR brachytherapy following NHT (which greatly varied) into two arms (≤0.1 ng/ml and >0.1 ng/ml) is shown in Fig. 3. The 5-year bRFS was 88.3% and 68.2%, respectively, showing a statistically significant difference between the two arms (*P =* 0.034).

On multivariate analysis, only the dose of HDR (*P* = 0.045, HR : 0.25, 95% CI : 0.038–0.97) and pre-HDR PSA level (*P* = 0.02, HR : 3.2, 95% CI : 1.18–10.16) were significant prognostic factors predicting bRFS (Fig. 3). On the other hand, age, i-PSA, Gleason score and duration of NHT were not statistically significant prognostic factors (*P* = 0.12, 0.47 and 0.66, respectively) (Table 3).

**Table 3.** Multivariate analysis by Cox proportional-hazard regression

Factor		*P*-value	Hazard risk	95% CI
i-PSA (ng/ml)	<20, ≥20	0.12	2.23	0.8–7.21
Gleason score	6–7 8 9–10	0.47	8/6–7:0.93 9–10/6–7:1.76 9–10/8:1.89	0.22–3.60 0.58–5.95 0.59–7.18
Duration of NHT (months)	≤6 >6, ≤12 >12	0.66	>6, ≤12/ ≤ 6:1.05 >12, ≤6:0.40 >12/ > 6, ≤12:0.42	0.35–2.98 0.02–2.65 0.02–2.7
Pre-HDR PSA (ng/ml)	≤0.1, >0.1	0.02	3.20	1.18–10.16
Dose of HDR BT (Gy)	11, 18	0.045	0.25	0.038–0.97

HR = Hazard Ratio. 95% CI = 95% Confidence Interval, i-PSA = initial PSA, NHT = Neoadjuvant Hormonal Therapy. HDR BT = High Dose Rate Brachytherapy.

## DISCUSSION

Radical radiotherapy for localized prostate cancer has been extensively studied. In particular, studies that have contributed to improvement in bRFS of prostate cancer are mainly classified into two categories. One category consists of studies on dose escalation for the prostate and seminal vesicle. These studies have led to a marked improvement in bRFS [1, 7–9], for which IMRT allowing dose escalation without increasing adverse events is essential. The other category consists of studies on concurrent hormonal therapy. In general, hormonal therapy (NHT) is not combined with surgery, because it has failed to improve long-term bRFS despite an increase in surgery patients with a negative margin [10]. As for radiotherapy, on the other hand, Bolla *et al.* [4] reported that conventional EBRT combined with 3-year AHT significantly improved bRFS [4]. Many Randomized Controlled Trials (RCTs) have been conducted to date, but combinations of NHT and AHT with varying durations were too complex to establish definite optimization of NHT. Among these RCTs, a Canadian trial is one of a few intended to optimize NHT, although AHT was not concomitantly used. Crook *et al.* reported the final results of the RCT comparing the 3-month NHT with the 8-month NHT [5].

According to a recent report from the Canadian trial [6], the PSA value (<0.1 ng/ml) before radiotherapy was the most important prognostic factor, irrespective of the duration (3 or 8 months) of NHT. The total dose level in the Canadian trial was 66–67 Gy (1.8–2.0 Gy/fx). Like those reported by Bolla *et al.* [4], these dose levels were relatively too low for high-risk prostate cancer without dose escalation, compared with current radical radiotherapy.

In the present study, some patients received HDR brachytherapy with dose escalation, and most patients received long-term AHT, which is generally standard treatment for high-risk prostate cancer. In univariate analysis, the dose level and the pre-HDR PSA were significant prognostic factors (*P* = 0.047 and 0.034, respectively) (Figs 1 and 3). At the same time, the dose of HDR (*P* = 0.045) and pre-HDR PSA level (*P* = 0.02) were significant prognostic factors predicting bRFS in multivariate analysis (Table 3). On the other hand, multivariate analysis did not identify the Gleason score as a significant factor, in disagreement with the Canadian trial [5, 6]. This might be explained by the fact that most of the patients in our study, unlike the Canadian trial, received 2-year AHT for high-risk prostate cancer, which reduced the difference between the two GS arms (6–7 and 8), however, further long-term follow-up will be required. The importance of dose escalation in high-risk prostate cancer has often been reported [1], and the dose level was a significant prognostic factor in our univariate and multivariate analysis.

As for the duration of NHT, the treatment outcome was worse in the ‘6 months < HT ≤ 12 months’ arm than expected. The cause remains unknown, since the percentage of patients with pre-HDR PSA ≤ 0.1 ng/ml and of those who received LDR brachytherapy was not unfavorable in this arm as compared with the ‘NHT ≤ 6 months’ arm. Liudgate *et al.* reported that the post-NHT PSA nadir, rather than the duration of the NHT, was important in NHT [11].

In the Canadian trial [5, 6], it was reported that the duration of the NHT was not a significant prognostic factor in the entire population, but this was not applicable to high-risk patients. In this regard, our finding may be consistent with that from the Canadian trial, since all patients in our analysis had high-risk prostate cancer.

Our retrospective study did not demonstrate i-PSA and GS as significant prognostic factors, although pre-HDR PSA and HDR dose were significant prognostic factors. Lower value of pre-HDR PSA is supposed to imply less tumor burden [12, 13]. It was reported that hormonal therapy resulted in an ‘oxygen effect’ by decreasing tumor burden [14–17], and also reported that hormonal therapy enhanced radiation-induced apoptosis in prostate cancer [18].

Movsas and Milosevic *et al.* reported that the tumor area was hypoxic in prostate cancer, and a tumor with low pO_2_ was associated with a higher risk of early PSA recurrence [14, 15]. While hypoxia may result in the activation of the angiogenesis pathway, Alonzi *et al.* demonstrated with dynamic MRI that androgen deprivation led to vascular collapse in prostate tumor [19]. Jain *et al.* suggested that hormonal agents may increase the percentage of normal vessels by inhibiting angiogenesis in hypervascular tumors, which results in oxygenation and thereby improvement in radiosensitivity [16]. It was also suggested that hormonal therapy may induce tumor shrinkage by stimulating apoptosis of tumor vascular endothelial cells [14–17].

Reducing tumor volume at the time of irradiation is considered to improve the effectiveness of radiotherapy, even if high doses are administered using HDR in our study. These things suggest that patients with a better response to NHT have shown more favorable prognosis regardless of the values of i-PSA. These results also correspond with the results of Zietman's report [20].

Coen *et al.* reported that high-risk prostate cancer (i-PSA > 15, GS ≥ 7, T3/4) was a more important prognostic factor for distant metastasis than other factors, and local recurrence was the most important prognostic factor for distant metastasis [21]. It is highly possible that NHT combined with HDR brachytherapy plus hypofractionated EBRT leading to dose escalation for the prostate and seminal vesicles may increase the local control rate and thereby prevent distant metastasis.

In radiotherapy for high-risk localized prostate cancer, concomitant long-term AHT has become standard treatment, but adequate dose escalation for the prostate and seminal vesicles as well as pre-treatment minimization of prostate cancer proliferation and tumor burden may be the most important thing, even if long-term AHT is combined.

In high-risk prostate cancer patients treated with the trimodality therapy (being composed of various treatments), our results confirmed the importance of the dose of HDR and the pre-HDR PSA nadir as prognostic factors. Especially, pre-HDR nadir PSA values of ≤0.1 ng/ml after NHT were related to improved bRFS. Further studies are needed to confirm the relevance of pre-HDR PSA nadir in trimodality therapy.

## CONFLICT OF INTEREST

This work was supported in part by a grant of SHIP0804 (NCT00664456).

## FUNDING

This work was supported in part by a grant of Foundation for Biomedical Research and Innovation (NCT00664456).
